# Novel *O*-GlcNAcylation on Ser^40^ of canonical H2A isoforms specific to viviparity

**DOI:** 10.1038/srep31785

**Published:** 2016-09-12

**Authors:** Mitsuko Hirosawa, Koji Hayakawa, Chikako Yoneda, Daisuke Arai, Hitoshi Shiota, Takehiro Suzuki, Satoshi Tanaka, Naoshi Dohmae, Kunio Shiota

**Affiliations:** 1 Laboratory of Cellular Biochemistry, Department of Animal Resource Sciences/Veterinary Medical Sciences, The University of Tokyo, Tokyo 113-8657, JAPAN; 2 Waseda Research Institute for Science and Engineering, Waseda University Tokyo 169-8555, JAPAN; 3 Biomolecular Characterization Unit, RIKEN Center for Sustainable Resource Science, Saitama 351-0198, JAPAN

## Abstract

We report here newly discovered *O*-linked-N-acetylglucosamine (*O*-GlcNAc) modification of histone H2A at Ser^40^ (H2AS40Gc). The mouse genome contains 18 H2A isoforms, of which 13 have Ser^40^ and the other five have Ala^40^. The combination of production of monoclonal antibody and mass spectrometric analyses with reverse-phase (RP)-high performance liquid chromatography (HPLC) fractionation indicated that the *O*-GlcNAcylation is specific to the Ser^40^ isoforms. The H2AS40Gc site is in the L1 loop structure where two H2A molecules interact in the nucleosome. Targets of H2AS40Gc are distributed genome-wide and are dramatically changed during the process of differentiation in mouse trophoblast stem cells. In addition to the mouse, H2AS40Gc was also detected in humans, macaques and cows, whereas non-mammalian species possessing only the Ala^40^ isoforms, such as silkworms, zebrafish and *Xenopus* showed no signal. Genome database surveys revealed that Ser^40^ isoforms of H2A emerged in Marsupialia and persisted thereafter in mammals. We propose that the emergence of H2A Ser^40^ and its O-GlcNAcylation linked a genetic event to genome-wide epigenetic events that correlate with the evolution of placental animals.

A wide functional and morphological diversity among the species is one of the features of the placenta^1^, reflecting the evolutionary history of the organ. Many placental genes are species-specific and are silenced in non-placental tissues^2^,^3^. However, most of the genes essentially involved in placental development are not expressed exclusively in the placenta^4^,^5^, suggesting that the original genes had already been established in a non-placental ancestor.

Histone modifications and DNA methylation are well-studied epigenetic mechanisms, which contribute to genome architecture and chromatin structure^6^,^7^,^8^,^9^. Epigenetic system underlies mammalian development, including the events of pre-implantation development and placentation^8^,^9^,^10^.

Core histone proteins are highly conserved throughout the animal kingdom^11^,^12^,^13^. The long term evolution of histones has been classically explained by a concerted evolution model, which accounts for the homogenization of the gene family members^14^,^15^. The canonical histone isoforms have been assumed to encode functionally equivalent proteins because only a few differences exist in their amino acid sequence^11^,^13^. However, it is not clear whether such small but distinct sequence variations have any influence on the biochemical character of the histone isoforms.

*O*-GlcNAcylation is a post-translational modification of proteins in which single *O*-GlcNAc moieties are attached to serine (Ser) or threonine (Thr) residues^16^,^17^,^18^. To date, 13 GlcNAcylated sites of core histones have been reported^16^,^17^,^19^, although their existence remains controversial. Earlier study describing most of the *O*-GlcNAcylated histone modifications had not been re-confirmed by mass spectrometry^20^. In addition, biological studies had been greatly impaired by the lack of the specific antibody.

Here, we report discovery of *O*-GlcNAcylated histone H2A at Ser^40^ (H2AS40Gc) by the combination of production of monoclonal antibody and the immunoprecipitation using wildtype and mutated recombinant H2A isoforms and mass spectrometric analyses with RP-HPLC fractionation.

## Results

### Characterization of monoclonal antibody 20B2 against *O*-GlcNAcylated Histone H2A

**Figure 1:**
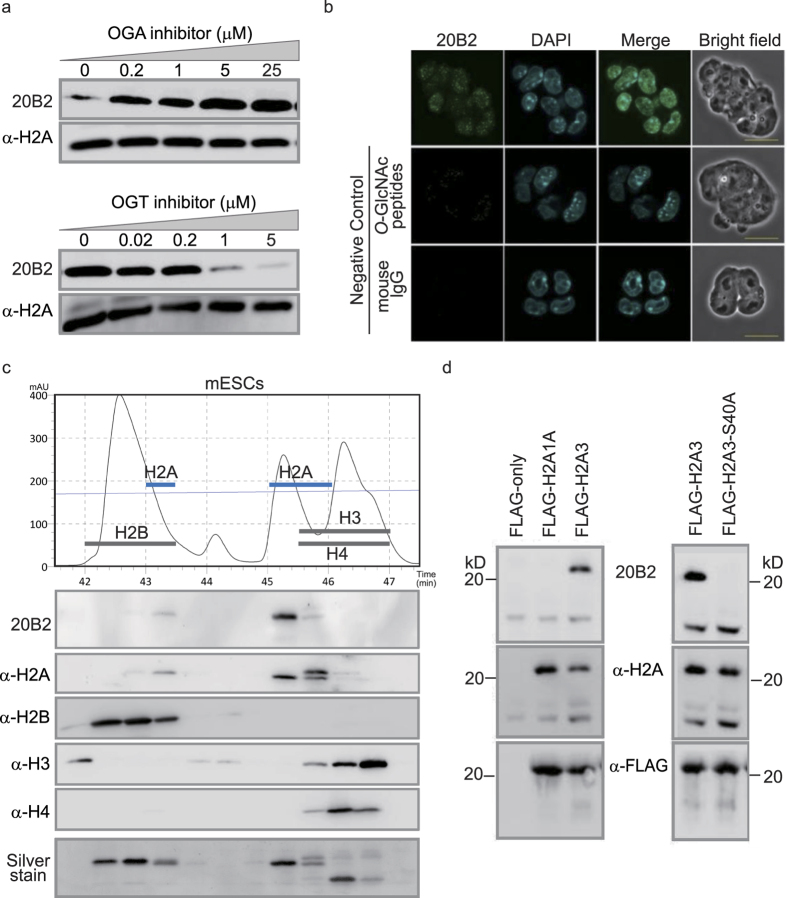
Characterization of monoclonal antibody 20B2 against *O*-GlcNAcylated Histone H2A. (**a**) WB by the monoclonal antibody (20B2) of mESC cell lysates treated with inhibitors of enzymes critical for *O*-GlcNAc modification. A pan-H2A antibody was used as a loading control. (**b**) IF of mESCs with 20B2 (green) and 4′, 6-diamidino-2-phenylindole (DAPI) (blue). Mouse IgG and 20B2 absorbed with *O*-GlcNAcylated peptides as negative controls. Scale bar = 20 μm. (**c)** Chromatogram, WB and Silver stain of separated mESC histones by RP-HPLC. Black line, protein retention; Thin blue line, acetonitrile gradient. Blotted with indicated antibodies. (**d**) IP of Flag-tagged H2A1A, H2A3, and H2A3-S40A mutants using anti-Flag antibody. Blotted with indicated antibodies.

To investigate *O*-GlcNAcylation of histones, we raised a monoclonal antibody (20B2) that reacts with *O*-GlcNAcylated peptides and epitopes of YT(Gc)E or YS(Gc)E sequences (Supplementary data Fig. 1a). Western blotting (WB) using 20B2 showed a single band (Supplementary data Fig. 1b,c) and its intensity was changed by *O*-GlcNAcase (OGA) or *O*-GlcNAc transferase (OGT) inhibitors in a dose-dependent manner (Fig. 1a). We observed the 20B2 signals as dotted foci in the nuclei of mouse embryonic stem cells (mESCs) by immunofluorescent staining (IF) (Fig. 1b). As reported by Shechter D. *et al*.^21^, RP- HPLC could separate each core histone into several fractions (Fig. 1c). The *O*-GlcNAcylation detected by 20B2 was limited to only some H2A isoforms.

Based on the Ensembl genome database (http://www.ensembl.org/index.html), there are 18 isoforms of canonical H2A in the mouse genome, of which 13 have Ser at position 40 (Ser^40^) (Supplementary data Fig. 1e). The other five H2A isoforms and H2A variants (H2AX, H2AZ, and H2Abad) have Ala^40^. We selected two H2A isoforms, H2A3 (*Hist3h2a*) for the Ser^40^ type and H2A1A (*Hist1h2aa*) for the Ala^40^ type, and prepared Flag-tagged recombinant proteins by transfecting mESCs. Immunoprecipitation (IP) and WB revealed that the Flag-H2A3, but not Flag-H2A1A, was recognized by 20B2. Furthermore, the reactivity was lost by the Ser^40^ to Ala^40^substitution (H2A3-S40A) (Fig. 1d).

### Mass spectrometry of G37-K74 peptides on H2A

**Figure 2:**
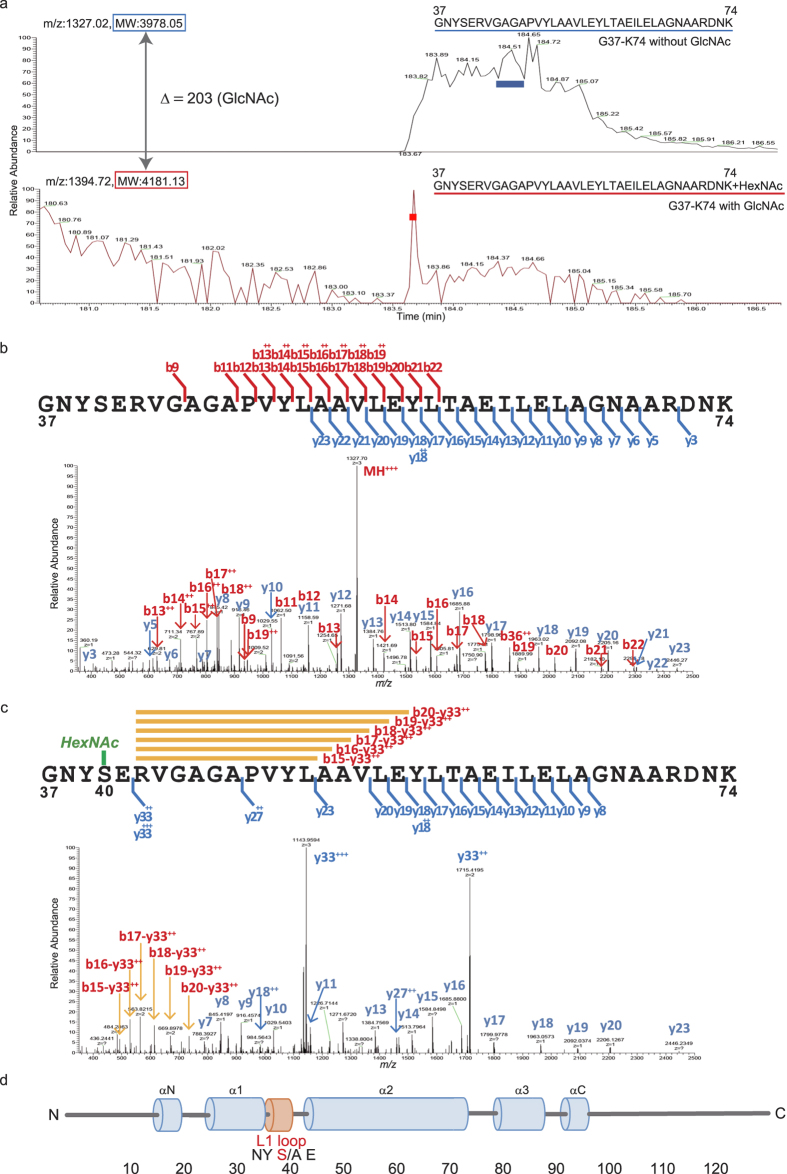
Mass spectrometry of G37-K74 peptides on H2A. (**a**) The selected ion chromatograms of triply charged G37-K74 peptide ion modified with (lower panel *m/z* 1394.72 shown in red: G37-K74 with GlcNAc) and without GlcNAc (upper panel *m/z* 1327.02 G37-K74 without GlcNAc) in the LC-MS of API digested of the 20B2-positive fraction (Fig. 1c) is indicated. (**b**) LC-MS/MS spectrum of triply charged G37-K74 without GlcNAc ion (*m/z* 1327.02). **(c**) LC-MS/MS spectrum of triply charged G37-K74 with GlcNAc ion (*m/z* 1394.72). (**d**) L1 loop of canonical histone H2A of the mouse. Blue cylinders, a-helical regions; orange cylinder, the L1 loop. The numbers indicate amino acid position.

20B2-positive fraction (Fig. 1c) was digested by *Achromobacter* protease I (API/Lys-C) and subjected to the liquid chromatography (LC)-tandem mass spectrometry (MS/MS). By LC-MS, we stably detected two peptide ions: G37-K74 peptides with (*m/z* 1394.72: G37-K74 with GlcNAc) or without modification (*m/z* 1327.02: G37-K74 without GlcNAc), supporting the concept that the G37-K74 peptide is modified by GlcNAc, according to the difference of theoretical MW (Δ = 203). Peaks of G37-K74 with and without GlcNAc (shown by red or blue bar respectively in Fig. 2a) were further analysed by higher-energy collisional dissociation (HCD) MS/MS (Fig. 2b,c). Compared with the spectrum of G37-K74 with or without GlcNAc, the doubly and triply charged y33 ions were observed specifically in a GlcNAc-modified peptide and internal ions of y33-b15 to y33-b20 were observed in a GlcNAc-modified peptide instead of b-series ions (Fig. 2b,c and Supplementary data Fig. 2). In a HCD MS/MS spectrum, peptide ions related to GlcNAc modification were hardly observed, so these spectra indicate that GlcNAc must be attached in the G37 to E41 region of the peptide. The cleavage of GlcNAc bound to S40 of the peptide ion in HCD mode is expected to affect the peptide bond cleavage of E41–R42 resulting in the y33 ions. Thus, 20B2 detects a newly discovered *O*-GlcNAc modification on the Ser^40^ of H2A isoforms located in the L1 loop of the globular domain, where interaction between the two H2As takes place^7^,^11^,^12^,^13^ (Fig. 2d).

The H2AS40Gc peptide was hardly detected by MS analysis using crude histone extracts without RP-HPLC fractionation. The GlcNAcylation of H2A Ser^40^ was also undetectable by other methods including WB with commercially available anti-*O*-GlcNAc antibodies (RL2 and CTD 110.6), wheat germ agglutinin, and Click-iT-Chemistry due to the limited amount of H2AS40Gc as previously reported^20^,^22^. The monoclonal antibody can clearly distinguish the presence or absence of the *O*-GlcNAc modification at the Ser^40^ of H2A isoforms (Supplementary data Fig. 1d) and will be useful for expanding the biological studies.

### Species-dependent *O*-GlcNAcylation of H2A isoforms

**Figure 3:**
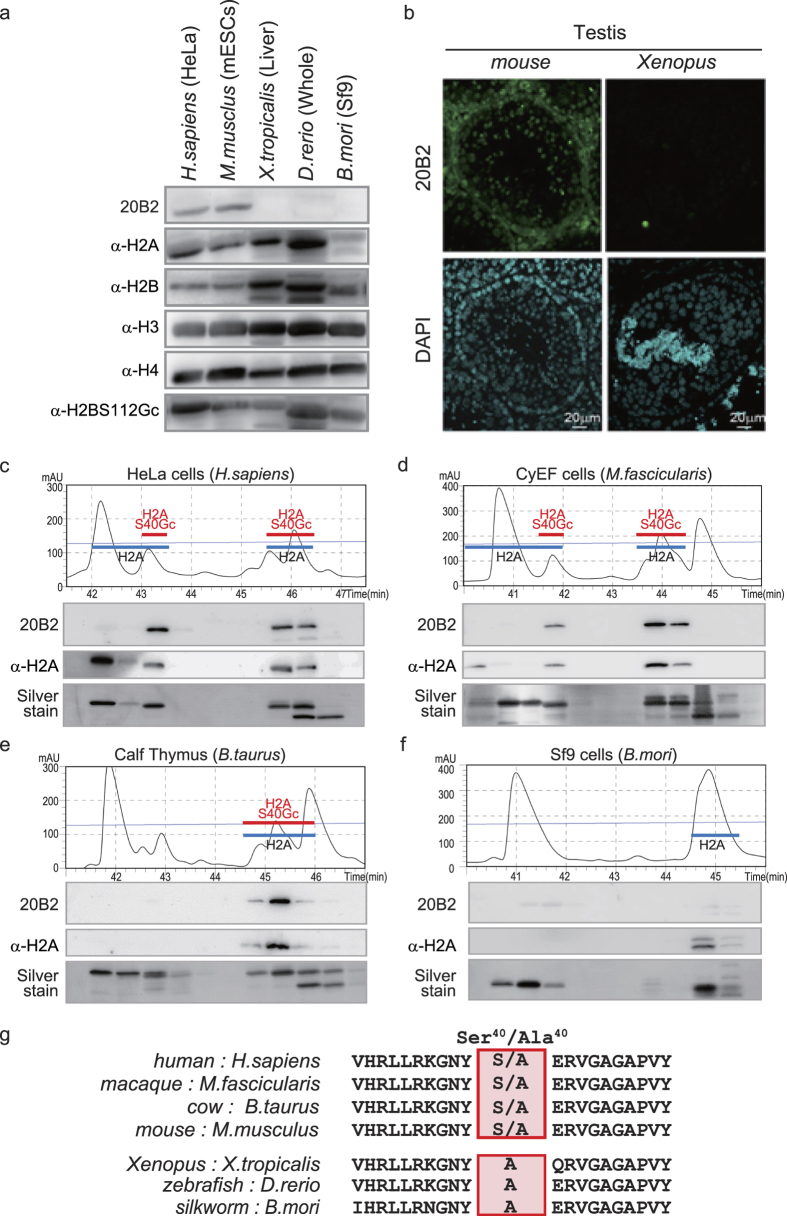
Species-dependent *O*-GlcNAcylation of H2ASer^40^. (**a**) WB by 20B2 in the crude histone fraction from various animals. (**b**) IF images of frozen testis sections from mouse and *Xenopus* with 20B2 (green) and DAPI (blue). (**c–f**) Chromatogram and WB of histones extracted from various animals. HeLa cells (human, (**c)**), CyEF cells (macaque, (**d**)), Calf thymus (cow, (**e**)), and Sf9 cells (silkworm, (**f)**). Black lines, protein retention; Thin blue lines, acetonitrile gradient. Each fraction was blotted with indicated antibodies and Silver stained. The fractions corresponding to H2A and H2AS40Gc were depicted. (**g**) Alignment of amino acid sequences around position 40 (boxed) of the canonical H2A of various animal species. Upper group has Ser^40^ or Ala^40^ and lower group has only Ala^40^.

We could detect H2AS40Gc in cells from mice and humans. However, it was undetectable in *Xenopus*, *Danio rerio*, or *Bombyx mori* (Fig. 3a). IF was performed on the testis of mice and *Xenopus*, based on the knowledge that the differentiation of male germ cells provides a large repertoire of histone markers^23^. In contrast to *Xenopus*, we detected H2AS40Gc signal in various cell types of mouse testis including spermatogonia (Fig. 3b). RP-HPLC analysis revealed that the chromatogram of silkworm cells is less complex than of other species we examined (Fig. 3c–f). Again, 20B2 did not give a positive signal with any fraction of the silkworm cells (Fig. 3f). Taken together, the *O*-GlcNAcylation of H2AS40 is specific to Mammalia, in contrast to that of H2BS112, which is conserved in metazoans^19^. It is noteworthy that the H2A isoforms of the animals with H2AS40Gc have Ser or Ala at position 40, while the H2AS40Gc-negative species only have Ala^40^ isoforms (Fig. 3g).

### Emergence of *O*-GlcNAcylated H2A isoforms in evolution of animals

**Figure 4:**
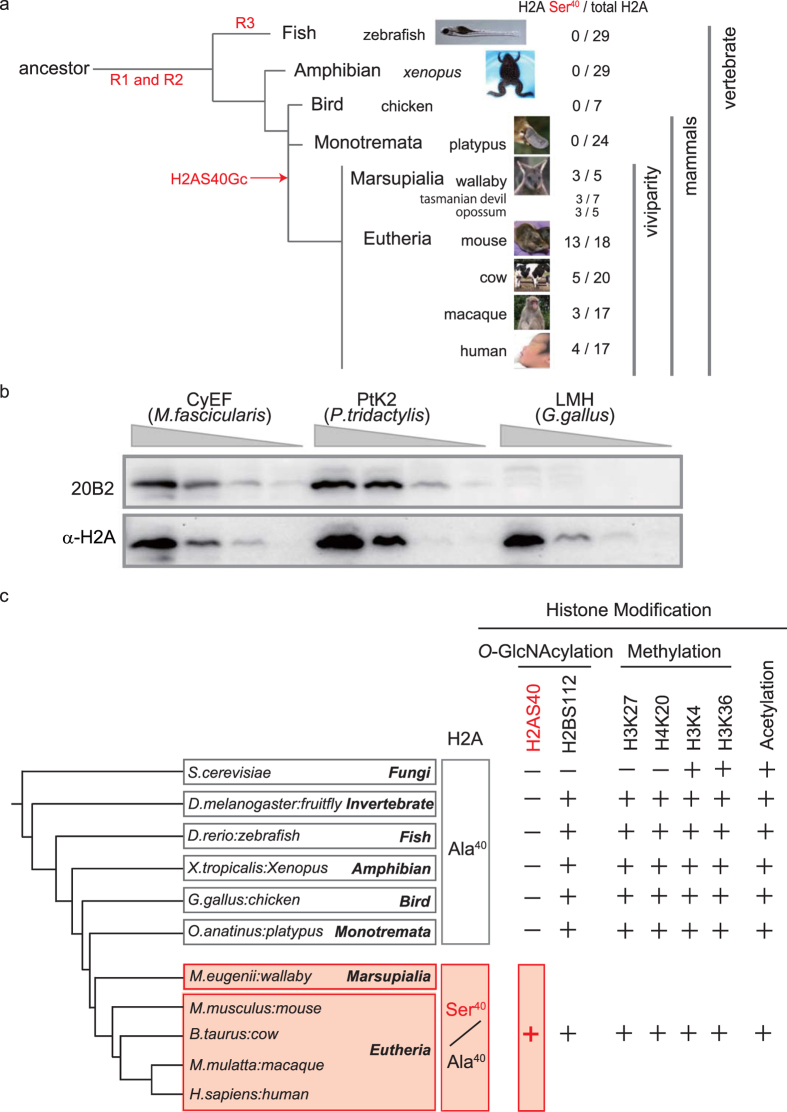
Emergence of Ser^40^ isoform of canonical H2A in animal evolution. (**a)** The numbers of Ser^40^ isoforms and all isoforms of H2A for indicated species are shown by a phylogenetic tree. R1-3, rounds of whole-genome duplication^15^. (**b**) WB of serial dilutions of crude histone extract from CyEF (macaque) cells, PtK2 (rat kangaroo) cells and LMH (chicken) cells with 20B2. A pan-H2A antibody was used as a loading control. (**c**) Representative histone modifications functioning as a part of the epigenetic mechanisms in animal species. −, not found; +, considered present.

In more than 70 animal species for which a full genome sequence is available from the Ensemble genome database, the Eutheria have the H2A isoforms with Ser^40^ (or at an equivalent position), whereas only Ala^40^ isoforms were found in Monotremata and non-mammalian animal species. A phylogenetic tree of H2A isoforms based on the amino acid sequences (Supplementary data Fig. 3) revealed isoform type-dependent clustering rather than a clustering based on species, which is a feature of the birth-and-death process of evolution^24^. Although copies of duplicated genes could be lost after the divergence in this evolutional process, the Ser^40^ isoform had not disappeared during marsupial and eutherian evolution, hinting at an evolutionary advantage of this isoform. The Ser^40^ ratio of total H2A isoforms is 3/5 in the wallaby, while it is 0/24 and 0/29 in the platypus and zebrafish, respectively (Fig. 4a), indicating that a simple increase in the copy number of the H2A gene does not account for the acquisition of a GlcNAcylation acceptor.

To verify whether or not H2AS40Gc exists in the Marsupials, PtK2 cells (rat kangaroo) were subjected to WB. The 20B2 antibody detected a band in a crude histone extract of PtK2, but not of LMH cells (chicken) (Fig. 4b). Whereas DNA methylation is not found, or is rare, in invertebrates, histone modifications are well conserved in both vertebrates and invertebrate, as shown in Fig. 4c^25^. In contrast, H2AS40Gc is an evolutionary-acquired histone modification in marsupial and placental animals (Fig. 4c and Supplementary data Fig. 5).

### Genome-wide distribution of H2AS40Gc

**Figure 5:**
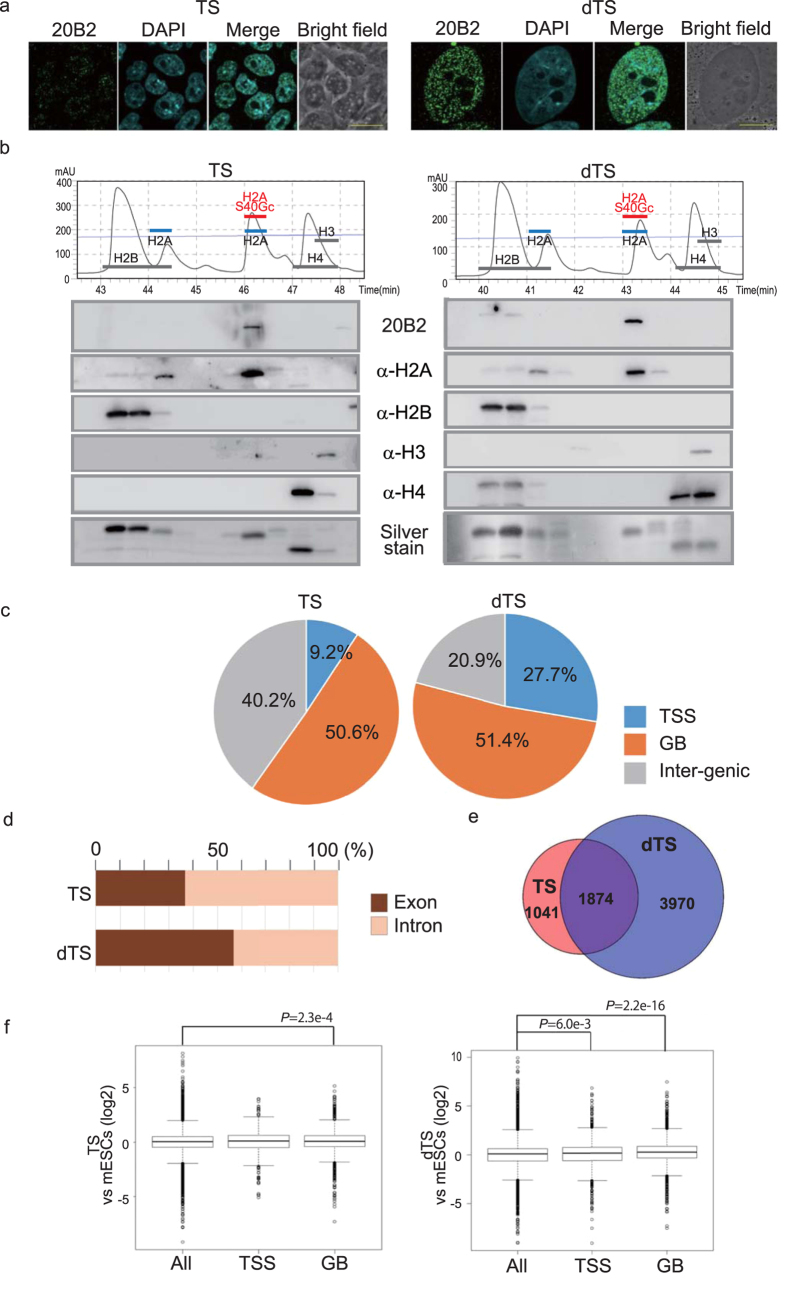
Genome-wide distribution of H2AS40Gc. (**a**) TS and dTS (day 6) were immunostained with 20B2 (green) and with DAPI (blue). Scale bar = 20 μm. (**b**) RP-HPLC chromatogram and WB of histones extracted from TS and dTS. (**c–f**) Summary of ChIP-seq analyses in TS and dTS. Distributions of H2AS40Gc peaks in TSS, GB and inter-genic regions (**c**), and in the distribution ratio exon and intron (**d**). (**e**) Venn diagrams showing the numbers of H2AS40Gc target genes in TS and dTS. (**f**) The box plots representing summary values of the expression of H2AS40Gc target genes in the TS and dTS relative to the ESCs.

The mouse trophoblast stem cells (TS) can differentiate *in vitro* to trophoblast subtypes (dTS), a majority of which consists of trophoblast giant cells^26^,^27^. An IF image showed an increase in the number and size of H2AS40Gc foci in the nucleus with differentiation (Fig. 5a). In RP-HPLC analysis, the H2A isoform component appeared similar between TS and dTS (Fig. 5b), suggesting that a differentiation-associated increase in H2AS40Gc is caused by accelerating GlcNAcylation rather than by changes in the composition of H2A isoforms.

The chromatin IP (ChIP)-seq analysis of TS and dTS using the 20B2 antibody (Supplementary data Fig. 4a,b) revealed a biased distribution of H2AS40Gc on the genome. Interestingly, more than 50% of the H2AS40Gc peaks were located at the gene body (GB) in both TS and dTS, while a shift to the transcription start site (TSS) (±2 kb of TSS) was obvious in dTS (Fig. 5c). Furthermore, the proportion of H2AS40Gc located within exons was more than 50% in dTS (Fig. 5d). Following differentiation of TS, the number of H2AS40Gc targets increased confirming the result of IF (Fig. 5e). Thus, the targets of H2AS40Gc were abundant, and were shifted by the process of differentiation. Analyses by ChIP-seq and expression array revealed that the genes with H2AS40Gc at GB in TS show relatively higher expression level compared with all genes, suggesting that H2AS40Gc at GB regulates the gene expression positively in TS. A similar positive correlation was also seen in the genes with H2AS40Gc at TSS or GB in dTS (Fig. 5f).

Gene ontology enrichment analysis of H2AS40Gc loci in dTS revealed a most significant enrichment of the term ‘organ morphogenesis’, implying a possible involvement of these genes in placentation (Supplementary data Fig. 4c). Interestingly, the Kyoto Encyclopedia of Genes and Genomes (KEGG) pathway analysis (http://www.genome.jp/kegg) of these loci highlighted Wnt signalling pathways, that has been shown to be a relevant regulator of trophectoderm lineage differentiation in human blastocysts^28^ (Supplementary data Fig. 4d). The pathways related to carcinoma or cancer^29^ is also enriched. The resemblance between dTS and cancer cells is often discussed, not only because of their invasive and migratory capacities, but also because they have many molecular circuits in common (Supplementary data Fig. 4c).

## Discussion

The H2AS40 is in the L1 loop of the histone globular domain, where two H2A molecules interact in the nucleosome^7^,^11^,^12^,^13^ (Supplementary data Fig. 5). H2AS40Gc may provide the variety in nucleosome structures. Consequently, the abundant H2AS40Gc targets distributed mainly in the gene body are expected to have an impact on the genome activity.

The hexosamine biosynthetic pathway produces UDP-GlcNAc, a substrate for *O*-GlcNAcylation, from glucose, glutamine, and acetyl CoA^17^,^18^. Therefore, *O*-GlcNAcylation of nuclear proteins including histones is considered as a nutrition sensing system that may regulate gene activity in response to the environment^1^,^18^. We suspect that H2AS40Gc may have provided a nutritional advantage for placental reproduction, although current data in this report are only correlative at the moment. It may be interesting to highlight the difference between H2AS40Gc signals in marsupial and eutherian placenta. The 20B2 antibody will be useful to analysis the invasive placenta of Eutherians.

Placental structure and function are prime examples of the complexity of adaptation^1^. Indeed, there are multiple variants among mammalian species. Studies of fish in the genus *Poeciliopsis* (Poeciliidae) suggest that adaptation of an intermediate stage of placentation is already found in non-placental animals^30^. During early vertebrate evolution, two rounds of whole-genome duplication (R1, R2) occurred, and another round (R3) was needed to achieve teleost lineage^15^ (Figure 4a). Because of the persistence of Ser^40^ isoforms of canonical H2A in Marsupials and Eutherians, we speculate that the emergence of the Ser^40^ isoform of canonical H2A and its *O*-GlcNAcylation had advantageous effect on the adaptation of viviparity. In the history of life, the acquisition of H2AS40Gc should have produced an explosive diversity of the chromatin structure through the variety of nucleosomes (Fig. 4c and Supplementary data Fig. 5).

## Methods

### Reagents, tissue preparation and cell culture

We purchased all reagents from Wako Pure Chemicals, unless otherwise stated.

Dr. Takayoshi Yamamoto and Dr. Masanori Taira (The University of Tokyo) provided livers from *Xenopus*. Dr. Hirokazu Enomoto and Dr Shinji Makino (Keio University) provided zebrafishes. We collected testis from adult *Xenopus* purchased from *Xenopus* Youshoku Kyozai (Ibaraki, Japan). We purchased adult C57BL/6Ncrj mice from Charles River Japan Inc. (Kanagawa, Japan). GenoStaff Co. (Tokyo, Japan) prepared cryosections of testes.

We obtained mESCs (J1 line) and HeLa cells from ATCC. Dr. Ikuhiro Okamoto (Kyoto University) kindly provided Cynomolgus monkey embryonic fibroblast (CyEF) cells. We obtained PtK2 cells and LMH cells, and Sf9 cells from JCRB (Osaka, Japan) and Gibco, respectively.

We cultured all cells according to the providers’ instructions at 37 °C in a humidified incubator (95% air, 5% CO_2_) unless otherwise stated. The mouse TS cells, previously derived from C57BL/6N blastocysts, were cultured as described elsewhere^31^. We cultured CyEF cells in the TS medium^31^ and Sf9 cells at 27 °C in an air incubator. We purchased benzyl 2-acetamido-2-deoxy-α-d-galactopyranoside (BADGP), an OGT inhibitor, from Sigma-Aldrich, and Thiamet-G, an OGA inhibitor, from Tocris. For inhibitor treatment, we cultured mESCs in the presence of BADGP or Thiamet-G at various concentrations for 96 hours.

Anti-H2A (ab13923), H2B (ab1790), H3 (ab1791), H4 (ab10158), and H2BS112Gc (ab130951) antibodies were purchased from Abcam. Anti-Flag M2 antibody (F1804) was purchased from Sigma-Aldrich. We used all of these antibodies according to the suppliers’ instructions.

### Generation of monoclonal antibody for H2AS40Gc (20B2)

Based on the preliminary MS analysis of the purified histones from mESCs, we selected several putative *O*-GlcNAcylation target sequences of the core histones (including GP537 and GP609, Supplementary data Fig. 1a). The Peptide Institute (Osaka, Japan) synthesized the polypeptides corresponding to these sequences with or without *O*-GlcNAc modification. Mice were immunized with an injection of a mixture of several *O*-GlcNAcylated synthetic peptides that were conjugated with recombinant GST and splenocyte hybridomas were prepared using a standard protocol at GenoStaff Co (Tokyo, Japan). The hybridomas were first screened by enzyme-linked immunosorbent assay (ELISA) with *O*-GlcNAcylated antigen peptides (GlcNAcylated peptides (GP), for positive screening) or with naked peptides with sequences identical to the antigen peptides (NP, for negative screening), and then verified by WB using crude histones (Supplementary data Fig. 1b,c) and ChIP using chromatin (Supplementary data Fig. 4a,b). The monoclonal antibody 20B2 was purified by affinity column of GP537 coupled Sepharose 4B (GE).

### Whole cell protein extraction and crude histone preparation

Whole-cell protein extracts were prepared using a RIPA buffer system (Santa Cruz Biotechnology). A Nuclear Extract Kit (Active Motif) and a LysoPure Nuclear and Cytoplasmic Extractor Kit were used for subcellular fractionation. A Histone Purification MINI Kit (Active Motif) was used to purify core histones. We performed all procedures according to the manufacturer’s instructions. We purchased calf thymus histones from Roche.

### WB analysis and Silver stain

Whole-cell lysates or crude histones were separated by sodium dodecyl sulfate - polyacrylamide gel electrophoresis (SDS-PAGE) on 20% acrylamide gel (XV Pantrea Gel, DRC, Tokyo, Japan).

For WB, separated proteins in gel were transferred to poly vinylidene difluoride membranes (Millipore), which were incubated overnight at 4 °C with 20B2 (1 μg/ml) in 5% bovine serum alubumin (BSA) and 0.1% Tween 20 in phosphate buffered saline (PBS). Horseradish peroxidase (HRP)-conjugated secondary goat anti-mouse IgG (Jackson Immuno Research) and Super Signal West Pico (Thermo) were used to detect immunoreactive proteins.

For absorbance experiments, 20B2 were pre-incubated with GP (1 μg/ml).

SilverQuest Silver Staining Kit (Invitrogen) was used according to the manufacturer’s instructions.

### Fractionation of core histones by RP-HPLC

Fractionation of crude histones was performed according to a procedure described by Shechter *et al*.^21^ with a slight modification. In brief, crude histones were dialyzed against Milli-Q water, and then the protein concentrations were determined using a BCA Protein Assay Kit following the manufacturer’s instructions. We separated crude histones (50 μg) using Aeris Widepore 3.6 μm XB-C8 column (Phenomenex) fitted to an LC-10Ai HPLC system (Shimadzu, Kyoto, Japan), that equilibrated with buffer A (5% acetonitrile, 0.1% trifluoroacetic acid (TFA)) and eluted with a linear gradient of 0%–0%–35%–35%–50% buffer B (90% acetonitrile, 0.1% TFA) over 0–5–15–25–62.5 min at a flow rate of 0.5 ml/min. We monitored elution by UV absorbance at 214 nm and subjected fractions to WB.

### Immunofluorescence staining of H2AS40Gc

Cells cultured on gelatin-coated glass coverslips were fixed with 4% paraformaldehyde followed by permeabilization with 0.2% Triton X-100. After blocking non-specific binding sites with 5% BSA, 0.1% Tween 20 in PBS for 1 h at room temperature, we incubated the fixed cells (1 μg/ml) or cryosections of testis (5 μg/ml) with 20B2 primary antibody overnight at 4 °C. We stained cells with AlexaFluor 488-conjugated goat anti-mouse IgG (Invitrogen) for 1 h at room temperature, followed by counterstaining with DAPI (1 μg/ml, Dojindo, Kumamoto, Japan) to visualize the nucleus and acquired confocal fluorescence images with an FV10i microscope (Olympus). As negative control, Mouse control IgG2a (1 μg/ml for fixed cells, 5 μg/ml for cryosections, Abcam, ab18413) were used. For absorbance experiments, the 20B2 antibody was pre-incubated with GP (1 μg/ml for fixed cells, 5 μg/ml for cryosections).

### Construction and transfection of overexpression vectors

Primers used for plasmid construction are listed in Supplementary data Table 1. DNA fragments for 3×Flag-tagged mouse *Hist3h2a* (encoding H2A3) and *Hist1h2aa* (encoding H2A1A) were generated by reverse transcription - polymerase chain reaction (RT-PCR) with total RNA of mESCs as a template using PrimeSTAR Max DNA Polymerase (TaKaRa), and cloned into the pENTR/D-TOPO vector (Invitrogen). Ser^40^ to Ala^40^ point mutation of H2A3 was introduced by PCR using the 3×Flag-H2A3 in pENTR/D-TOPO vector as a template. We used BigDye sequencing (Applied Biosystems) to confirm the sequences of resulting constructs.

Using Gateway LR Clonase (Invitrogen), the 3×Flag-tagged cDNAs were subcloned into a pCAG-DEST-pA-PGK-Puromycin-IRES-Venus vector^32^ for mammalian overexpression or into a pET301 vector (Invitrogen) for bacterial expression.

### Recombinant proteins

We cultured mESCs in 6-well plates under stem cell conditions and then transfected the cells with 2 μg of plasmid and 4 μl of jetPRIME reagent (Polyplus-tranfection) per well. We re-fed cells with fresh medium containing 5 μg/ml puromycin 24 h after the transfection, and incubated them for another 48 h until harvesting and preparation of insoluble nuclear fractions using a LysoPure Nuclear and Cytoplasmic Extractor Kit.

pET301 3×Flag-tagged mouse *Hist3h2a* was transformed into Rosetta 2 bacteria (Novagen). Following induction with 1 mM IsoPropyl b-D-1-ThioGalactopyranoside, the bacterial pellet was resolved in lysis buffer (40 mM Tris-HCl pH 7.5, 5 mM ethylenediamine tetraacetic acid (EDTA), 0.5% Triton X-100) and incubated on ice for 30 min. We sonicated the suspension and centrifuged it at 20,000 g for 30 min to obtain supernatant used following purification.

For purification of 3×Flag-tagged mouse *Hist3h2a* recombinant proteins, mESCs insoluble nuclear fraction or bacterial lysate were applied using anti-FLAG M2 Magnetic Beads (Sigma-Aldrich) according to the manufacturer’s instructions.

### Immunoprecipitation

For immunoprecipitation of 3×Flag-tagged H2A isoforms, insoluble nuclear fractions were collected from transfected cells using a LysoPure Nuclear and Cytoplasmic Extractor Kit. Proteins (100 μg each) were mixed with 20 μl of anti-FLAG M2 Magnetic Beads (Sigma-Aldrich) and incubated with rotation at 4 °C overnight. Precipitates were washed five times with TBS, and then subjected to SDS-PAGE followed by WB.

### ChIP-seq

ChIP was performed with 1 × 10^7^ cells of TS or dTS at confluent in 10 cm dish per assay using a ChIP-IT Express Enzymatic Kit (Active Motif) according to the manufacturer’s instructions with minor modifications. Briefly, fixed cells were lysed and mixed with an enzymatic shearing cocktail for 10 min (TS) or 5 min (dTS). The sheared chromatin (100 μl) was mixed with 3 μg of 20B2 antibody and 40 μl of Dynabeads M-280 sheep anti-mouse IgG (Invitrogen), and then incubated with rotation at 4 °C overnight. After IP, DNA was recovered by incubation in the elution buffer (10% SDS, 300 mM NaCl, 10 mM Tris-HCl, and 5 mM EDTA, pH 8.0) at 65 °C for 6 h. Recovered DNA was purified using a ChIP DNA Clean and Concentration Kit (Zymo Research).

The ends of 50 ng of DNA fragments isolated by ChIP were repaired using T4 DNA polymerase (New England Biolabs) and phosphorylated with T4 polynucleotide kinase (New England Biolabs). A single “A” base was added to the 3′-end with Klenow fragments (New England Biolabs). TruSeq DNA adapters (Illumina) were ligated to the fragments with DNA ligase (New England Biolabs). Ligation products between 200 and 600 bp were purified on AMpure XP beads (Beckman Coulter Inc.) to remove unligated adapters and subjected to 14 cycles of PCR amplification. Completed libraries were quantified using an Agilent 2000 BioAnalyzer system. The DNA libraries were analysed using an Illumina Hiseq 2000 system (The Center for Epigenomics at the Albert Einstein College of Medicine).

We determined the read quality of each sample using FastQC software. After pre-filtering the raw data by removing sequence adapters and low-quality reads, we mapped the tags to the mouse genome (assembly mm9) using Bowtie software and detected peaks using MACS software from a Galaxy browser (www.galaxy.psu.edu). We used the following parameter settings: input-seq aligned reads as a control file, 100 bp tag size, and 125 bp bandwidth. Venn diagram analysis was performed with Venny 2.0 software (http://bioinfogp.cnb.csic.es/tools/venny/). We deposited ChIP-seq data in the Gene Expression Omunibus database under accession number GSE74342. Other pulished expression array data. GSM325436 (TS), GSM325442 (dTS) and GSM 902302 (ES). were used for analysis.

### Mass spectrometry

The HPLC-purified fraction was neutralized with Tris-HCl buffer, pH 8.0, followed by digestion with *Achromobacter* protease I (API/Lys-C)^33^ (a gift from Dr. Takeharu Masaki (Ibaraki University)). Then, digest was analysed by nanoliquid chromatography–tandem mass spectrometry (nLC-MS/MS) using a Q-Exactive mass spectrometer (Thermo). We separated the peptides using a nano ESI spray column (75 μm × 100 mm, NTCC analytical column C18, 3 μm, Nikkyo Technos, Tokyo, Japan) that was equilibrated with buffer A (0.1% aqueous formic acid) and eluted with a linear gradient of 5%–5%–15%–40%–60% buffer B (0.1% formic acid in 100% acetonitrile) over 0–10–120–180–220 min at a flow rate of 300 nL/min (Easy nLC, Thermo). We operated the mass spectrometer in a positive-ion mode, and the MS and MS/MS spectra were acquired using a data-dependent TOP10 method. We searched the MS/MS spectra against an in-house database using a local Mascot server (version 2.5, Matrix Sciences). The triply charged GlcNAc peptide ion of *m*/*z* 1394.7 was confirmed using a targeted MS/MS mode.

### ELISA

We coated 96-well ELISA plates with 100 μl/well of various synthetic peptides (5 μg/ml) or purified Flag-tagged recombinant H2A3 (ES or *Escherichia coli* (*E. coli*)) at 37 °C for 1 h. After blocking non-specific binding sites with 5% BSA and 0.1% Tween 20 in PBS for 1 h at room temperature, we incubated plates with 50 μl/well of 20B2 primary antibody (1 μg/ml) overnight at 4 °C. We then washed the plates with PBS and incubated them with HRP-conjugated anti-mouse IgG secondary antibodies (Jackson ImmunoResearch) diluted at 1:10000 for 1 h at room temperature. After washing three times with PBS, we added OPD substrate in 1× stable peroxide substrate buffer (Thermo) and read the absorbance at 490 nm using an iMark microplate reader (Bio-Rad). For Flag-tagged recombinant H2A3 assay, we used an anti-Flag M2 antibody (Sigme-Aldrich, F3165) as a primary antibody for normalization of H2A. We assayed each sample in duplicate and expressed the results as the mean of two readings.

### Phylogenetic tree construction

The whole-genome sequence data were obtained from the Ensembl Genome Browser (http://www.ensembl.org/index.html). Phylogenetic trees were constructed using the MAFFT multiple sequence alignment program (http://www.ebi.ac.uk/Tools/msa/mafft/).

### Species pictures

Some species images were downloaded from http://www.genome.gov. (zebrafish credited by Shawn Burgess, NHGRI, platypus by Nicole Duplaix, Getty Images, wallaby by Courtesy State of Victoria (Australia), Department of Innovation, Industry and Regional Development, and mouse by Maggie Bartlett, NHGRI).

## Additional Information

**Accession codes:** ChIP-seq data has been deposited in the Gene Expression Omnibus database under accession number GSE74342.

**How to cite this article**: Hirosawa, M. *et al*. Novel *O*-GlcNAcylation on Ser^40^of canonical H2A isoforms specific to viviparity. *Sci. Rep.* **6**, 31785; doi: 10.1038/srep31785 (2016).

## Supplementary Material

Supplementary Information
