# Supplementary material for: Novel O-GlcNAcylation on Ser40 of canonical H2A isoforms specific to viviparity
Source: Sci Rep. 2016 Sep 12;6:31785. doi: 10.1038/srep31785 (PMC5018834; doi:10.1038/srep31785)

Title

Novel *O*-GlcNAcylation on Ser^{40} of canonical H2A isoforms specific to viviparity.

Authors

Mitsuko Hirosawa*, Koji Hayakawa*, Chikako Yoneda, Daisuke Arai, Hitoshi Shiota, Takehiro Suzuki, Satoshi Tanaka, Naoshi Dohmae, Kunio Shiota

(*These anuthors contributed equally to this work)

Supplementary Figure legends

Supplementary data Figure 1. Antigen specificity assay of the 20B2 monoclonal antibody and a phylogeny of H2A isoforms.

a, Synthetic peptides with (GP) or without (NP) *O*-GlcNAcylation were subjected to ELISA using 20B2 as a primary antibody. **b,** WB analysis of mESC whole-cell lysates with 20B2. There is a 20B2-positive band (left lane). The 20B2 band was vanished by pre-incubation with GP (1 μg/ml) (right lane). **c,** WB analysis with 20B2 (left lane) and silver stained (right lane) crude histone extracts of mESCs. **d,** Purified Flag-tagged recombinant H2A3 produced by mESC (ES-FLAG H2A3, blue) was subjected to ELISA using 20B2 as a primary antibody. We used anti-FLAG antibody to normalize H2A3 levels. *E. coli* recombinant proteins (*E. coli*-FLAG H2A3, orange) were used for negative control of *O*-GlcNAc modification. **e,** Amino acid sequence-based phylogeny of genes for mouse canonical H2A isoforms. The amino acid at position 40 (Ser^{40}or Ala^{40}) and the gene cluster numbers are shown in the right margin.

Supplementary data Figure 2. Mass spectrometry of G37-K74 peptide on H2A.

Summary of the theoretical values of MS/MS fragments of G37-K74 peptide. The observed ions in Fig. 2b and 2c are written in red.

Supplementary data Figure 3. Phylogeny of H2A isoforms of animals.

Phylogenetic relationship of canonical H2A proteins in selected animal species (human, macaque, cow, horse, rat, mouse, wallaby, platypus, chicken, frog or *Xenopus*, zebrafish, and fruit fly). The amino acid at position 40 (Ser^{40} or Ala^{40}) and the cluster numbers of genes for respective H2A isoforms are shown in the right margin.

Supplementary data Figure 4. ChIP-seq analysis of TS and dTS.

a, As the validation of 20B2 for ChIP, mESC chromatin (an equivalent amount of 1 μg genomic DNA) and 3 μg of 20B2 were used. The 20B2-positive band (IP) was vanished when 20B2 was pre-incubated with GP （IP+GP, 3 μg GP）, but not with NP (IP+NP, 3 μg GP). Mouse IgG as a negative control. **b,** Shearing check of chromatin used in ChIP-seq. There was no long chromatin in both TS and dTS samples. **c,** The gene ontology enrichment analysis of biological processes and KEGG pathway analysis of genes harbouring H2AS40Gc. Top five categories for each analysis of TS-specific and dTS-specific target genes. **d,** H2AS40Gc target genes in dTS related to the Wnt signal pathway (red boxes).

Supplementary data Figure 5. Schematic model for the acquisition of H2A Ser^{40} *O*-GlcNAcylation in the evolutionary process. The emergence of the Ser^{40} isoform of canonical H2A and its *O*-GlcNAcylation occurred at the time of the acquisition of placenta. In the histone octamer, two H2A molecules interact at the L1 loop of the globular domain where S40 can be *O*-GlcNAcylated. H2AS40Gc may produce complexity of the chromatin through the variety of nucleosomes.

Supplementary data Table 1. Primer lists. For construction of 3xFlag-fused H2A3 and H2A3-mutant overexpression vectors [a-e], same alphabet indicate that primer is used as the combination.

Supplementary Information. Uncropped images with size maker indications

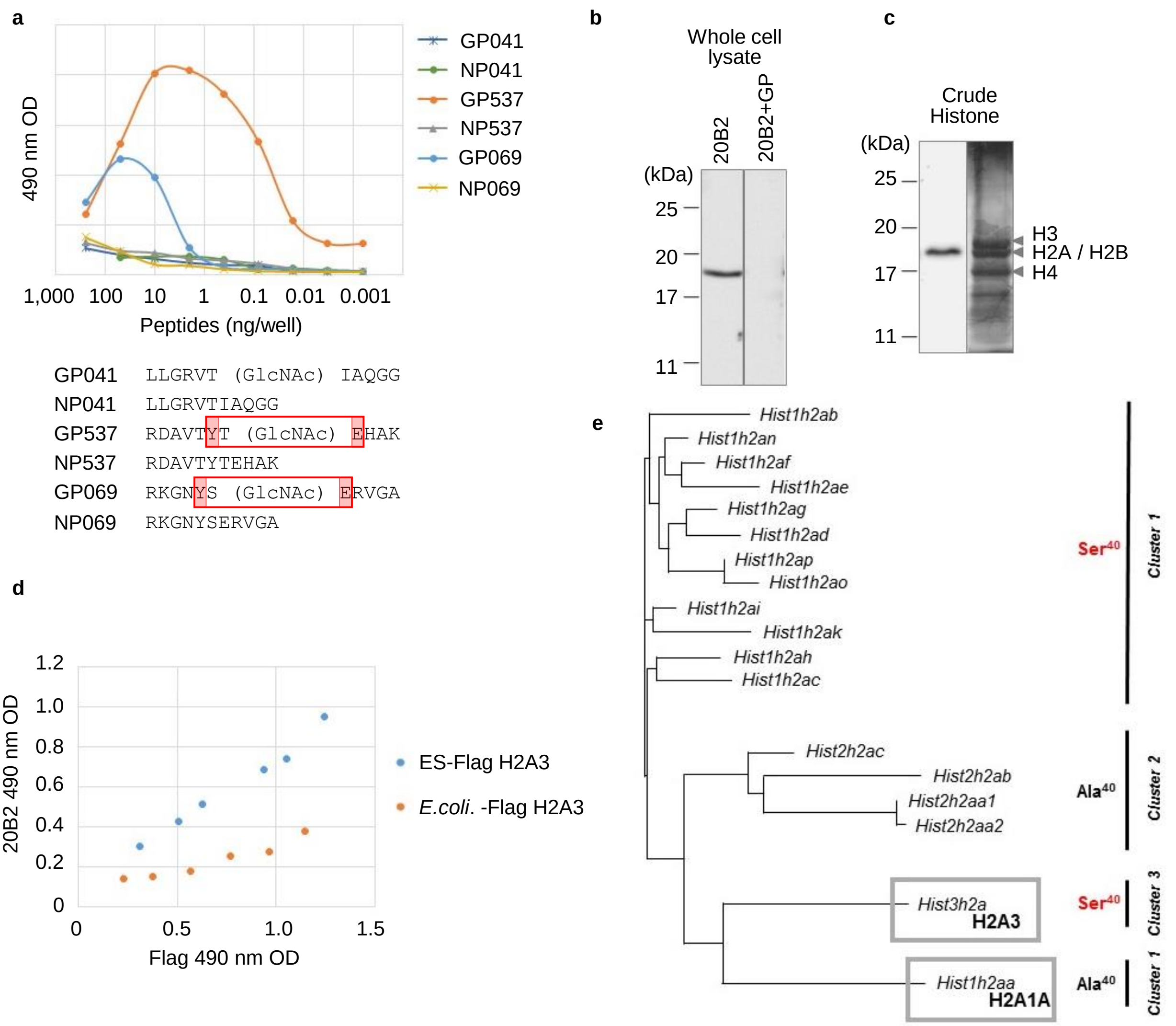

Supplementary data Figure 1. Antigen specificity assay of the 20B2 monoclonal antibody and a phylogeny of H2A isoforms. a, Synthetic peptides with (GP) or without (NP) *O*-GlcNAcylation were subjected to ELISA using 20B2 as a primary antibody. **b,** WB analysis of mESC whole-cell lysates with 20B2. There is a 20B2-positive band (left lane). The 20B2 band was vanished by pre-incubation with GP (1 µg/ml) (right lane). **c,** WB analysis with 20B2 (left lane) and silver stained (right lane) crude histone extracts of mESCs. **d ,** Purified Flag-tagged recombinant H2A3 produced by mESC (ES-FLAG H2A3, blue) was subjected to ELISA using 20B2 as a primary antibody. We used anti-FLAG antibody to normalize H2A3 levels. *E. coli* recombinant proteins (*E. coli*-FLAG H2A3, orange) were used for negative control of *O*-GlcNAc modification. **e,** Amino acid sequence-based phylogeny of genes for mouse canonical H2A isoforms. The amino acid at position 40 (Ser^{40}or Ala^{40}) and the gene cluster numbers are shown in the right margin.

MS/MS fragmentation of GNYSERVGAGAPVYLAAVLEYLTAEILELAGNAARDNK

b	b++	Res			y"	y++
58.029	29.518	1	Gly	38	-	-
172.072	86.54	2	Asn	37	3922.04	1961.52
335.136	168.071	3	Tyr	36	3807.99	1904.5
422.168	211.587	4	Ser	35	3644.93	1822.97
551.21	276.109	5	Glu	34	3557.9	1779.45
707.311	354.159	6	Arg	33	3428.85	1714.93
806.38	403.693	7	Val	32	3272.75	1636.88
863.401	432.204	8	Gly	31	3173.69	1587.35
934.438	467.723	9	Ala	30	3116.66	1558.84
991.46	496.234	10	Gly	29	3045.63	1523.32
1062.5	531.752	11	Ala	28	2988.61	1494.81
1159.55	580.278	12	Pro	27	2917.57	1459.29
1258.62	629.813	13	Val	26	2820.52	1410.76
1421.68	711.344	14	Tyr	25	2721.45	1361.23
1534.77	767.886	15	Leu	24	2558.38	1279.7
1605.8	803.405	16	Ala	23	2445.3	1223.15
1676.84	838.923	17	Ala	22	2374.26	1187.64
1775.91	888.458	18	Val	21	2303.23	1152.12
1888.99	945	19	Leu	20	2204.16	1102.58
2018.04	1009.52	20	Glu	19	2091.07	1046.04
2181.1	1091.05	21	Tyr	18	1962.03	981.518
2294.18	1147.6	22	Leu	17	1798.97	899.987
2395.23	1198.12	23	Thr	16	1685.88	843.445
2466.27	1233.64	24	Ala	15	1584.84	792.921
2595.31	1298.16	25	Glu	14	1513.8	757.402
2708.39	1354.7	26	Ile	13	1384.76	692.881
2821.48	1411.24	27	Leu	12	1271.67	636.339
2950.52	1475.76	28	Glu	11	1158.59	579.797
3063.6	1532.31	29	Leu	10	1029.54	515.276
3134.64	1567.82	30	Ala	9	916.46	458.734
3191.66	1596.34	31	Gly	8	845.423	423.215
3305.71	1653.36	32	Asn	7	788.401	394.704
3376.74	1688.88	33	Ala	6	674.359	337.683
3447.78	1724.39	34	Ala	5	603.321	302.164
3603.88	1802.44	35	Arg	4	532.284	266.646
3718.91	1859.96	36	Asp	3	376.183	188.595
3832.95	1916.98	37	Asn	2	261.156	131.082
-	-	38	Lys	1	147.113	74.06

b	b++	Res			y	y++	y+++
58.029	29.518	1	Gly	38	-	-	-
172.072	86.54	2	Asn	37	4125.11	2063.06	1375.71
335.136	168.071	3	Tyr	36	4011.07	2006.04	1337.7
625.247	313.127	4	Ser-HexNAc	35	3848.01	1924.51	1283.34
754.29	377.648	5	Glu	34	3557.9	1779.45	1186.64
910.391	455.699	6	Arg	33	3428.85	1714.93	1143.62
1009.46	505.233	7	Val	32	3272.75	1636.88	1091.59
1066.48	533.744	8	Gly	31	3173.69	1587.35	1058.57
1137.52	569.262	9	Ala	30	3116.66	1558.84	1039.56
1194.54	597.773	10	Gly	29	3045.63	1523.32	1015.88
1265.58	633.292	11	Ala	28	2988.61	1494.81	996.873
1362.63	681.818	12	Pro	27	2917.57	1459.29	973.194
1461.7	731.352	13	Val	26	2820.52	1410.76	940.843
1624.76	812.884	14	Tyr	25	2721.45	1361.23	907.82
1737.85	869.426	15	Leu	24	2558.38	1279.7	853.466
1808.88	904.945	16	Ala	23	2445.3	1223.15	815.771
1879.92	940.463	17	Ala	22	2374.26	1187.64	792.092
1978.99	989.997	18	Val	21	2303.23	1152.12	768.413
2092.07	1046.54	19	Leu	20	2204.16	1102.58	735.39
2221.11	1111.06	20	Glu	19	2091.07	1046.04	697.696
2384.18	1192.59	21	Tyr	18	1962.03	981.518	654.681
2497.26	1249.13	22	Leu	17	1798.97	899.987	600.327
2598.31	1299.66	23	Thr	16	1685.88	843.445	562.632
2669.35	1335.18	24	Ala	15	1584.84	792.921	528.95
2798.39	1399.7	25	Glu	14	1513.8	757.402	505.271
2911.47	1456.24	26	Ile	13	1384.76	692.881	462.256
3024.56	1512.78	27	Leu	12	1271.67	636.339	424.562
3153.6	1577.3	28	Glu	11	1158.59	579.797	386.867
3266.68	1633.85	29	Leu	10	1029.54	515.276	343.853
3337.72	1669.36	30	Ala	9	916.46	458.734	306.158
3394.74	1697.88	31	Gly	8	845.423	423.215	282.479
3508.79	1754.9	32	Asn	7	788.401	394.704	263.472
3579.82	1790.42	33	Ala	6	674.359	337.683	225.458
3650.86	1825.93	34	Ala	5	603.321	302.164	201.779
3806.96	1903.98	35	Arg	4	532.284	266.646	178.1
3921.99	1961.5	36	Asp	3	376.183	188.595	126.066
4036.03	2018.52	37	Asn	2	261.156	131.082	87.724
-	-	38	Lys	1	147.113	74.06	

Supplementary data Figure 2. Mass spectrometry of G37-K74 peptide on H2A . Summary of the theoretical values of MS/MS fragments of G37-K74 peptide. The observed ions in Fig. 2b and 2c are written in red.

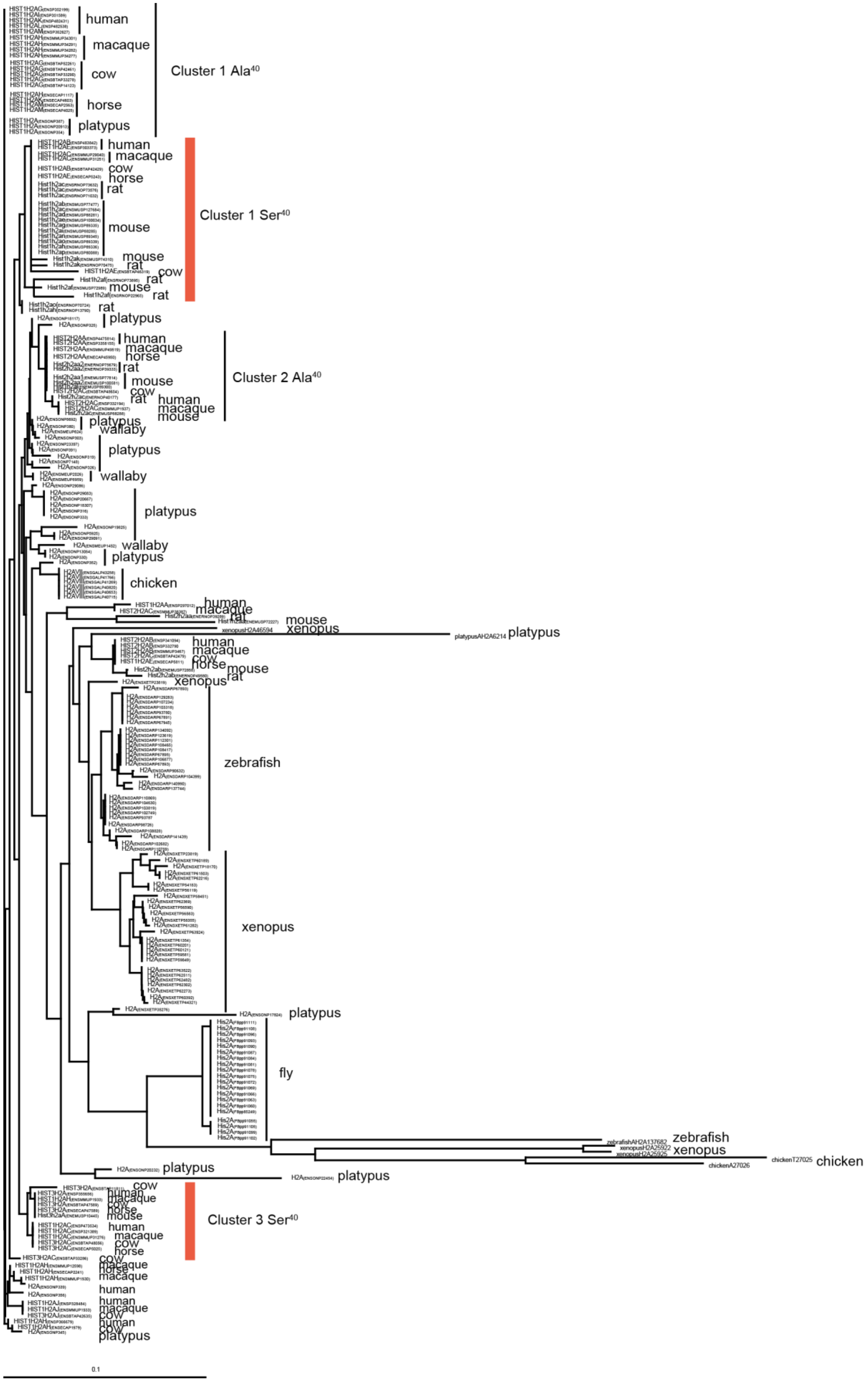

Supplementary data Figure 3. Phylogeny of H2A isoforms of animals. Phylogenetic relationship of canonical H2A proteins in selected animal species (human, macaque, cow, horse, rat, mouse, wallaby, platypus, chicken, frog or *Xenopus*, zebrafish, and fruit fly). The amino acid at position 40 (Ser^{40} or Ala^{40}) and the cluster numbers of genes for respective H2A isoforms are shown in the right margin.

a

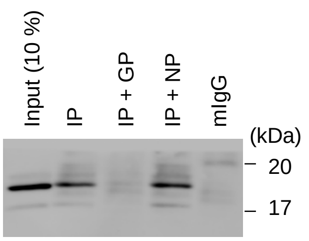

b

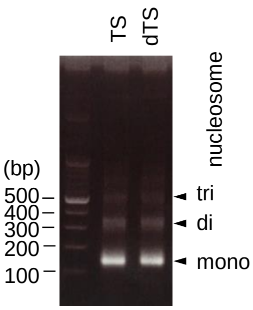

c

Annotation of TS-specific targets

GO analysis (BP)	P-value
Cation transport	7.7E-04
Cellular amine metabolic process	2.1E-03
Negative regulation of cell communication	5.5E-03
Cellular amino acid derivative biosynthetic process	9.8E-03
Regulation of neurological system process	1.2E-02
**Pathway analysis (KEGG)**	**P-value**
Axon guidance	3.7E-03
Neuroactive ligand-receptor interaction	4.7E-02
Glycerophospholipid metabolism	6.9E-02
Adipocytokine signaling pathway	6.9E-02
Long-term potentiation	8.0E-02

Annotation of dTS-specific targets

GO analysis (BP)	P-value
organ morphogenesis	2.7E-12
regulation of gene expression	2.0E-11
negative regulation of macromolecule biosynthetic process	1.1E-07
central nervous system development	3.0E-09
positive regulation of nucleobase, nucleoside, nucleotide and nucleic acid metabolic process	8.6E-07
**Pathway analysis (KEGG)**	**P-value**
Wnt signaling pathway	1.2E-07
Hedgehog signaling pathway	1.2E-05
Basal cell carcinoma	1.7E-05
Calcium signaling pathway	2.3E-05
Pathways in cancer	6.1E-05

d

Wnt signaling pathway

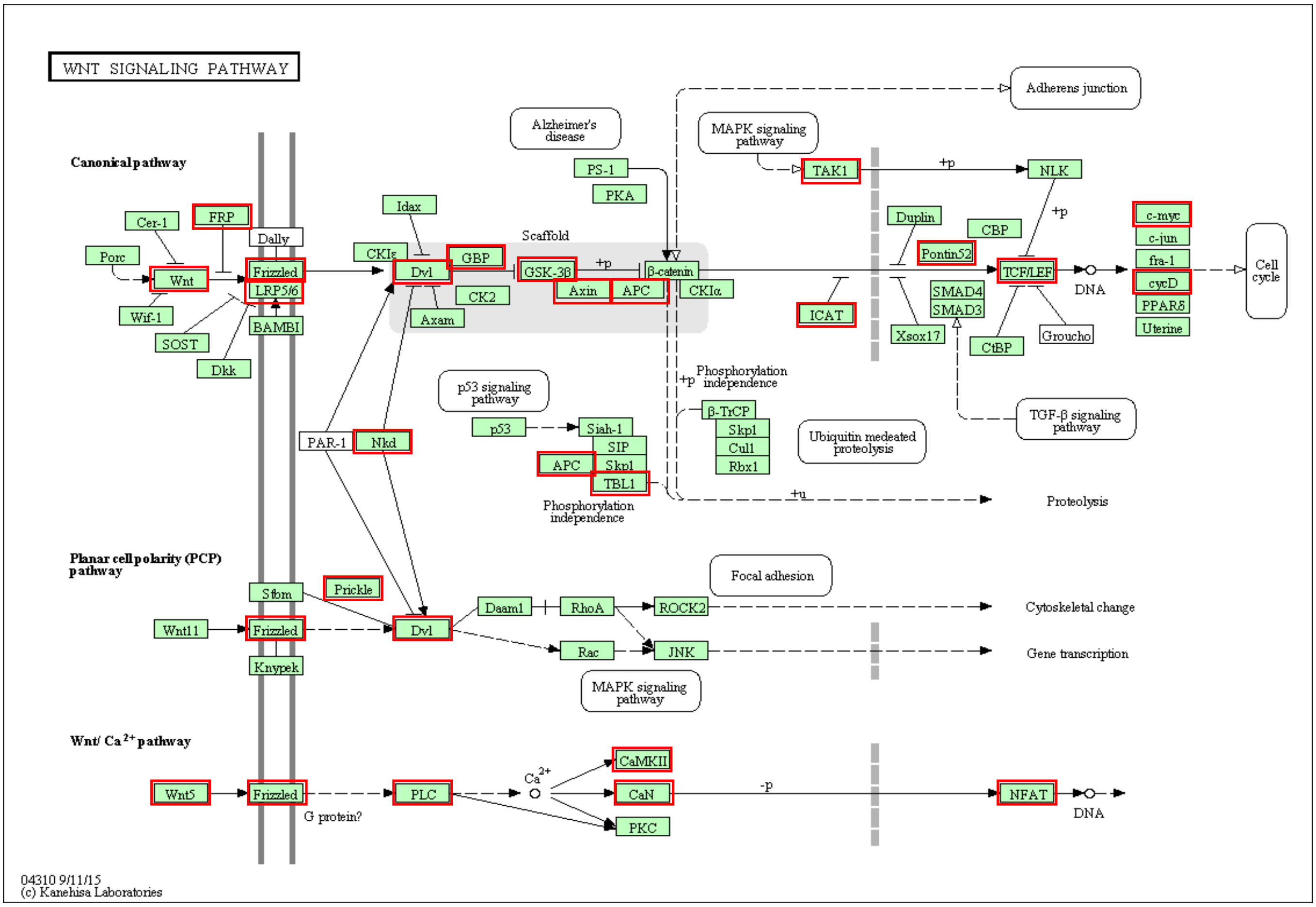

Supplementary data Figure 4. ChIP-seq analysis of TS and dTS. a, As the validation of 20B2 for ChIP, mESC chromatin (an equivalent amount of 1 µg genomic DNA) and 3 µg of 20B2 were used. The 20B2-positive band (IP) was vanished when 20B2 was pre-incubated with GP（IP+GP, 3 µg GP）, but not with NP (IP+NP, 3 µg GP). Mouse IgG as a negative control. **b,** Shearing check of chromatin used in ChIP-seq. There was no long chromatin in both TS and dTS samples. **c,** The gene ontology enrichment analysis of biological processes and KEGG pathway analysis of genes harbouring H2AS40Gc. Top five categories for each analysis of TS-specific and dTS-specific target genes. **d,** H2AS40Gc target genes in dTS related to the Wnt signal pathway (red boxes).

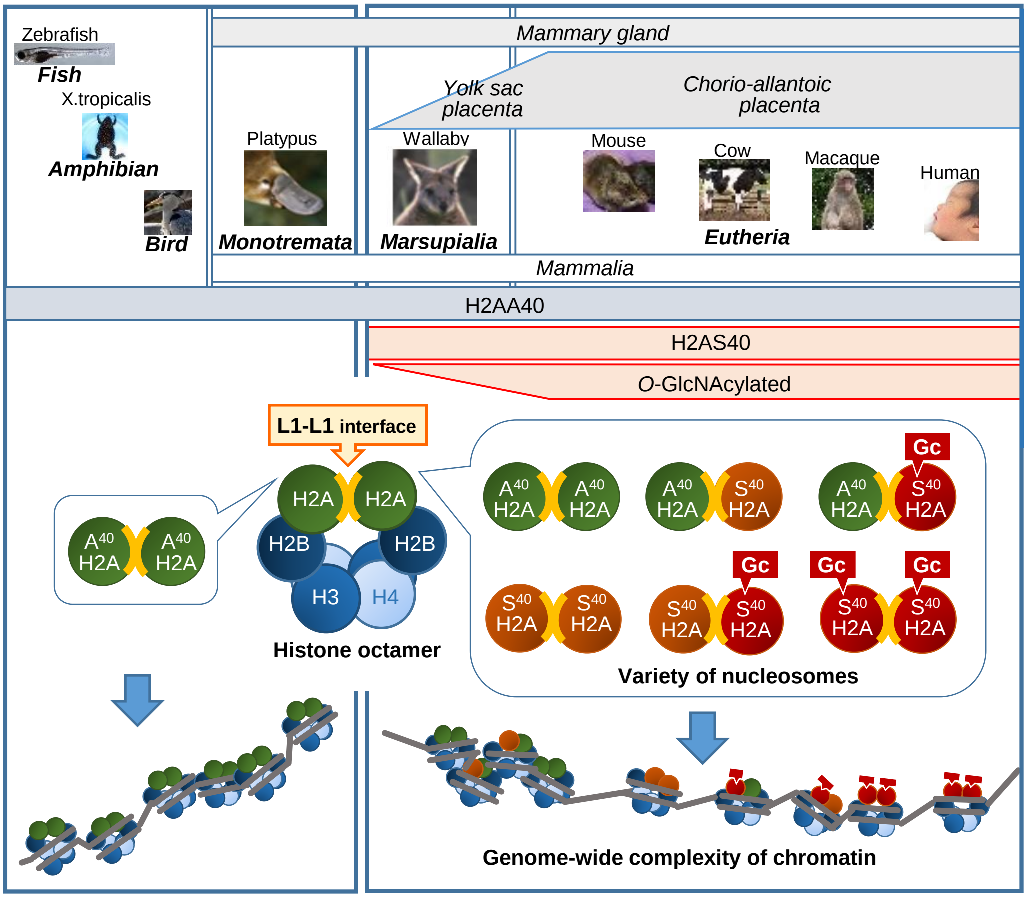

Supplementary data Figure 5. Schematic model for the acquisition of H2A Ser^{40} *O*-GlcNAcylation in the evolutionary process. The emergence of the Ser^{40} isoform of canonical H2A and its *O*-GlcNAcylation occurred at the time of the acquisition of placenta. In the histone octamer, two H2A molecules interact at the L1 loop of the globular domain where S40 can be *O*-GlcNAcylated. H2AS40Gc may produce complexity of the chromatin through the variety of nucleosomes.

Primer name	Sequence
**3xFlag_H2A3_F1**[a]	AAAGATCATGATATCGATTACAAGGATGACGATGACAAGATGTCTGGTCGTGGCAAGCAGGGCG
**3xFlag_H2A3_R**[a, c]	AAGAGCCTTTGGTGTCTGTAAAAGTC
**3xFlag_F2**[c, d]	CACCATGGACTACAAAGACCATGACGGTGATTATAAAGATCATGATATCGATTACAAGG
**3xFlag_R**[d]	CTACTTGTCATCGTCATC
**H2A3-S40A_F**[e]	AATTATTCGGAGCGGGTGGGCGCTGGT
**H2A3-S40A _R**[e]	CCGCTCCGAATAATTACCCTTACGGAG

Supplementary data Table 1. Primer lists. For construction of 3xFlag-fused H2A3 and H2A3-mutant overexpression vectors [a-e], same alphabet indicate that primer is used as the combination.

Supplementary Information. Uncropped images with size maker indications

Fig. 1a

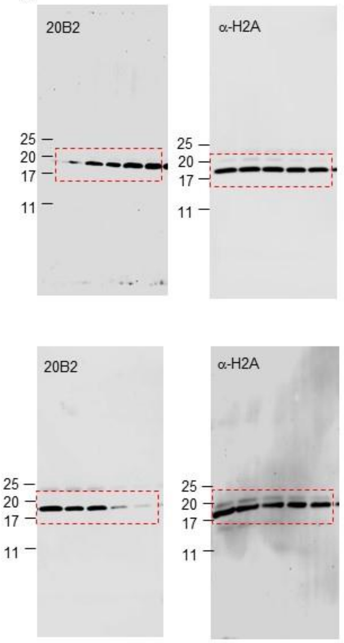

Fig. 1c

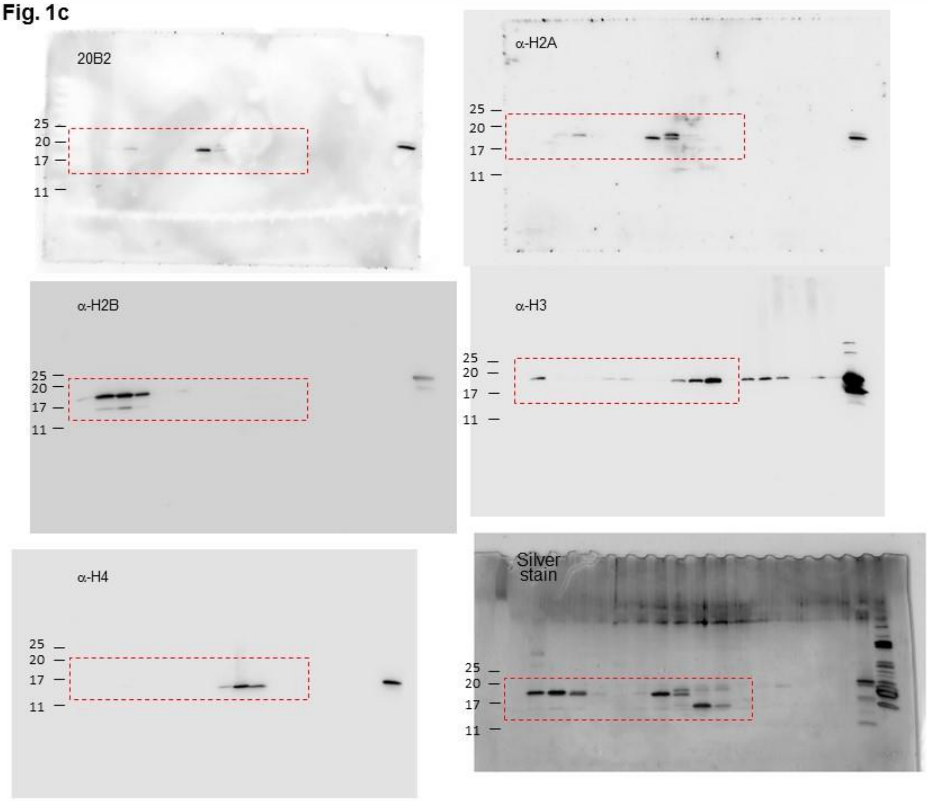

Fig. 1d

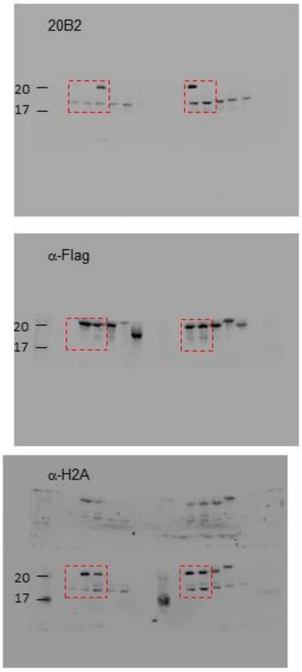

Fig. 2a

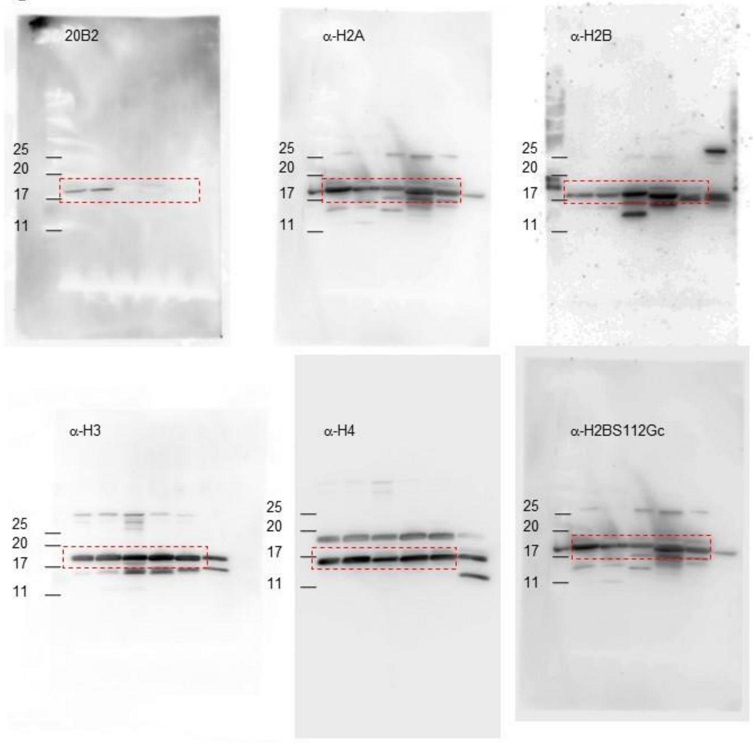

Supplementary Information. Uncropped images with size maker indications

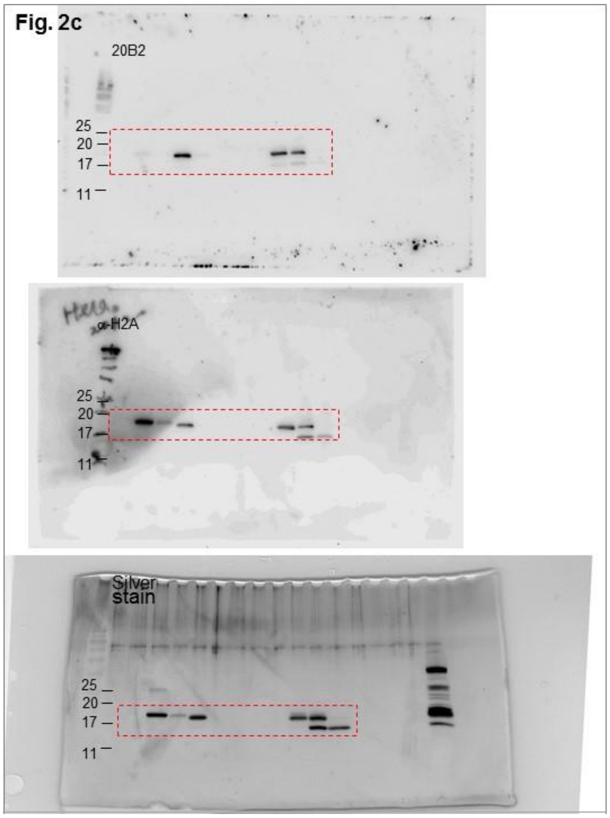

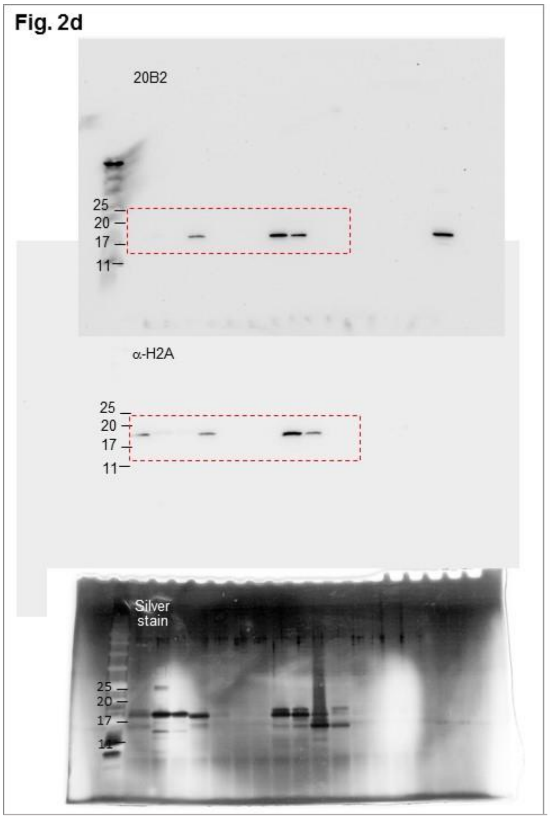

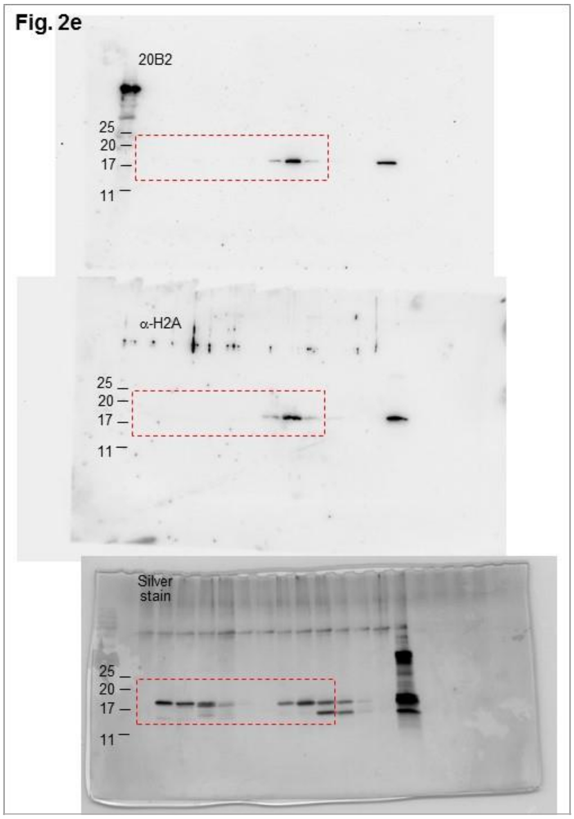

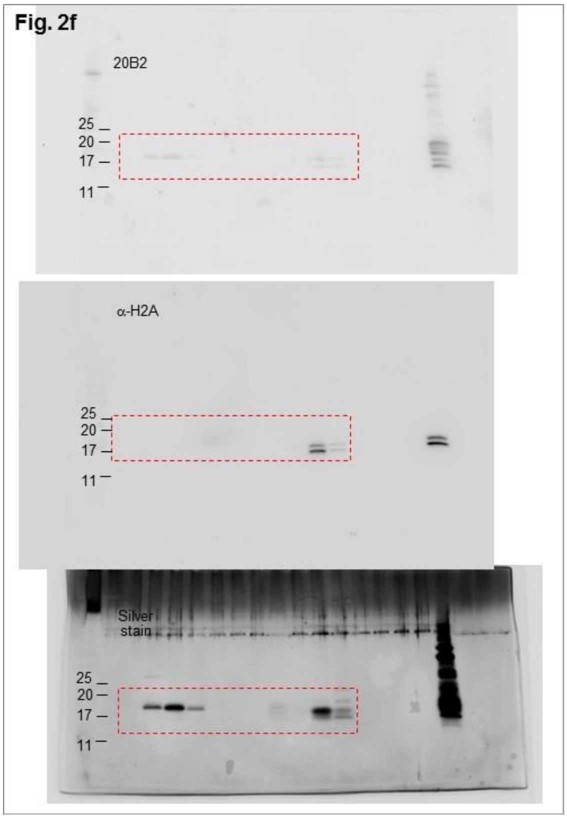

Supplementary Information. Uncropped images with size maker indications

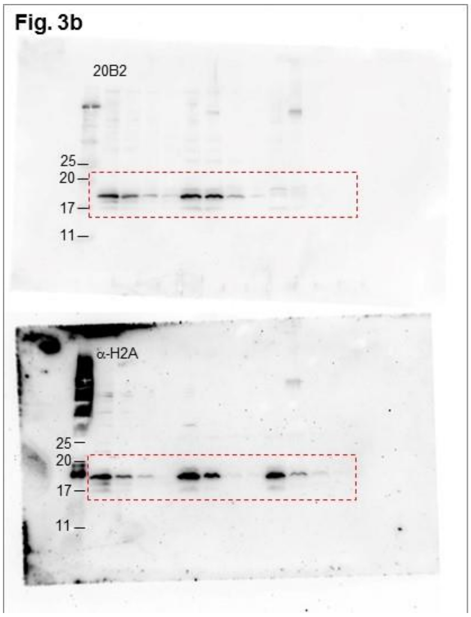

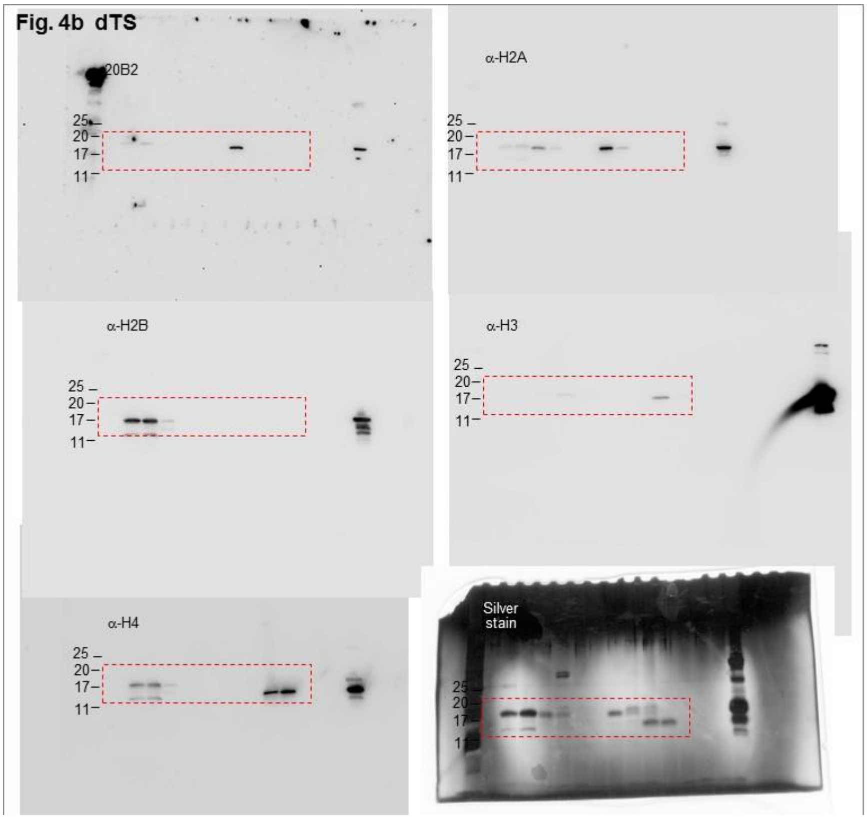

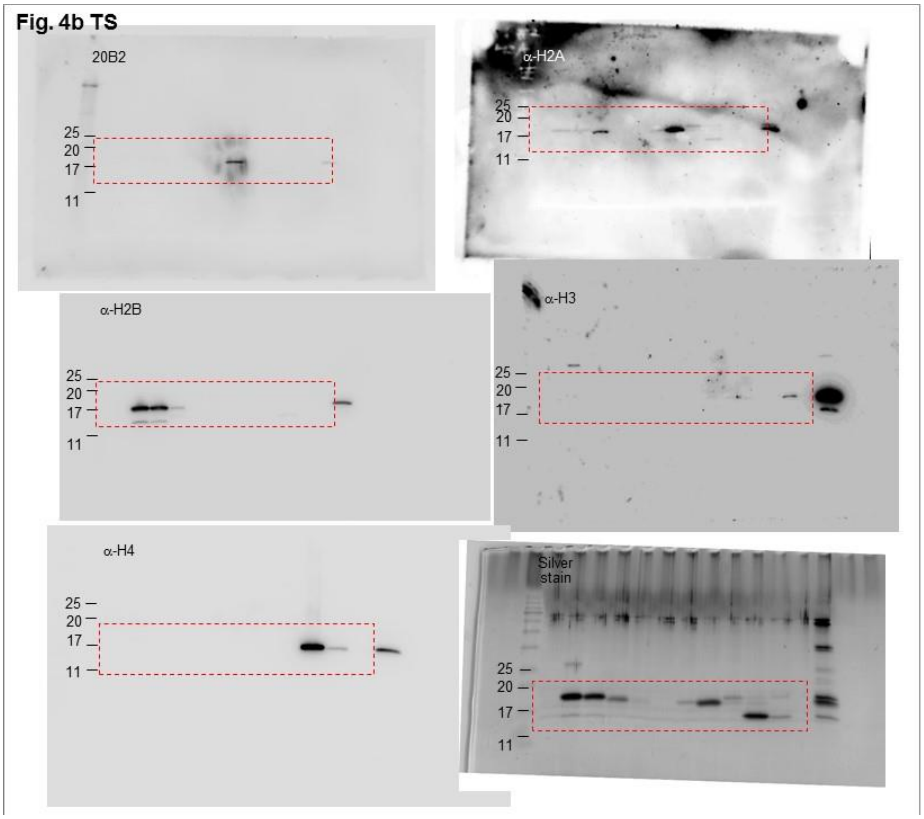

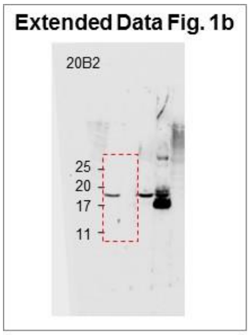

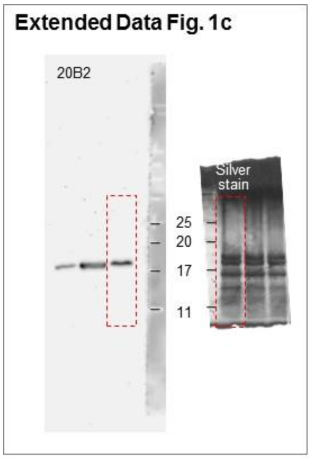

Supplementary Information. Uncropped images with size maker indications

Extended Data Fig. 4a

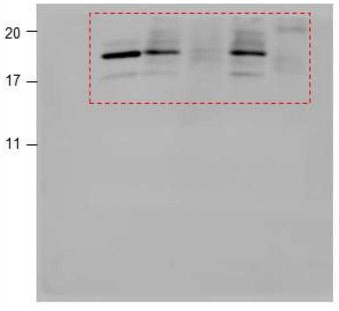

Extended Data Fig. 4b

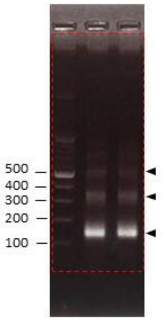

Supplement: Supplementary Information [file srep31785-s1.pdf]
